# Efficacy of interstitial photodynamic therapy using talaporfin sodium and a semiconductor laser for a mouse allograft glioma model

**DOI:** 10.1038/s41598-024-59955-y

**Published:** 2024-04-21

**Authors:** Kenta Nagai, Jiro Akimoto, Shinjiro Fukami, Yuki Saito, Emiyu Ogawa, Masakatsu Takanashi, Masahiko Kuroda, Michihiro Kohno

**Affiliations:** 1 Department of Neurosurgery, Tokyo Medical University, 6-7-1 Nishishinjuku, Shinjuku-Ku, Tokyo, 160-0023 Japan; 2 Faculty of Science and Technology, Keio University, Kanagawa, Japan; 3 Department of Molecular Pathology, Tokyo Medical University, Tokyo, Japan

**Keywords:** Interstitial photodynamic therapy, Talaporfin sodium, Apoptotic cell death, Vascular shutdown, Light delivery, Surgical oncology, CNS cancer

## Abstract

To investigate the therapeutic potential of photodynamic therapy (PDT) for malignant gliomas arising in unresectable sites, we investigated the effect of tumor tissue damage by interstitial PDT (i-PDT) using talaporfin sodium (TPS) in a mouse glioma model in which C6 glioma cells were implanted subcutaneously. A kinetic study of TPS demonstrated that a dose of 10 mg/kg and 90 min after administration was appropriate dose and timing for i-PDT. Performing i-PDT using a small-diameter plastic optical fiber demonstrated that an irradiation energy density of 100 J/cm^2^ or higher was required to achieve therapeutic effects over the entire tumor tissue. The tissue damage induced apoptosis in the area close to the light source, whereas vascular effects, such as fibrin thrombus formation occurred in the area slightly distant from the light source. Furthermore, when irradiating at the same energy density, irradiation at a lower power density for a longer period of time was more effective than irradiation at a higher power density for a shorter time. When performing i-PDT, it is important to consider the rate of delivery of the irradiation light into the tumor tissue and to set irradiation conditions that achieve an optimal balance between cytotoxic and vascular effects.

## Introduction

Glioblastoma is the most intractable of the primary brain tumors. Factors that determine the prognosis include patient factors such as age and pretreatment performance status, as well as treatment factors such as the extent of resection by craniotomy and postoperative radiation therapy and chemotherapy^1–3^. In other words, the first step of treatment is to perform a maximum safe resection to remove as much of the tumor as possible without worsening the patient’s neurological symptoms. In 2001, Lacroix. et al.^4^ stated that removal of more than 98% of the tumor bulk significantly increases the patient’s survival period. However, it was not easy to achieve safe resections of 98% or more of the tumor bulk at a time when intraoperative navigation systems and physiological monitoring systems were not yet widely available. In 2011, Sanai. et al.^5^ discussed the importance of preserving brain function while maintaining the goal of total resection, pointing out the importance of performing surgeries on patients in whom more than 78% of the tumor bulk can be removed. However, the results of a phase III trial conducted by Stummer et al.^6^, who established the fluorescence guide resection technique showed that this method improves the extent of tumor resection and increases the median progression-free survival time (m-PFS), but does not increase the median overall survival time (m-OS). In other words, the problem of glioblastoma cell infiltration, which is not captured by imaging, is the main cause of the difficulty of tumor control, even if the tumor appears to have been removed completely on imaging^7^. Therefore, in addition to maximum safe resection, the control of invasive cells is important to improve the prognosis of patients with glioblastoma.

Radiotherapy and chemotherapy using the alkylating agent temozolomide after tumor resection have been the global standard of care to control cell infiltration of glioblastoma, but most tumors recur locally within about 7 months, and the m-OS of patients is only about 15 months^8^. To date, several treatment modalities have been developed to improve the prognosis of this refractory tumor in combination with standard therapies^9^. Novel therapies such as BCNU wafers, which aim to kill invasive tumor cells by infiltrating the resection cavity with anticancer agents^10^; bevacizumab, which controls neovascularization that feeds residual tumor cells^11^; and tumor-treating fields, which control the growth of residual tumor cells^12^, have been approved and used in clinical practice worldwide, but have not shown sufficient success to be effective in improving the outcome of patients with glioblastoma^9–12^.

To overcome this situation, the authors have pursued the possibility of additional intraoperative photodynamic therapy (PDT)^13^. This therapy is a combined therapy using a photosensitizer (PS), which selectively accumulates in tumor cells, and a laser beam that can excite the PS^13,14^. The principle of this therapy is that the PS accumulating in tumor cells and the energy generated during the photochemical reaction by the excitation laser light convert the oxygen dissolved in tumor cells into highly toxic singlet oxygen, resulting in cellular-level killing effects^14^. The original PS used worldwide, was the hematoporphyrin derivatives (HpD), but there were problems with its low tissue accumulation and expensive excitation laser equipment, as well as its characteristic induction of strong skin photosensitivity reactions^13^. Nevertheless, clinical studies have been conducted in Scotland^15^, Canada^16^, Australia^17^, Germany^18^, Austria^19^, and elsewhere, and have shown potential for the use of HpD in the treatment of glioblastoma. On the other hand, we have been analyzing the possible application of talaporfin sodium (mono-l-aspartyl chlorine e6: TPS), a chlorin derivative synthesized in Japan, as a PS since 2000^13^. We have confirmed the selective uptake and retention of TPS using cultured glioma cells and animal glioma models, and have demonstrated the efficacy of PDT for these using laser light that can excite TPS in many experimental studies^20–23^. Based on these proofs of concept, we conducted a clinical trial at our institution^24^, and a physician-initiated clinical trial of PDT with TPS for primary malignant brain tumors was initiated in 2009^25^. The results demonstrated a favorable effect of PDT with TPS for patients with newly diagnosed glioblastoma with a m-PFS of 12 months and a m-OS of 24.8 months, as well as high safety, and PDT using TPS was approved for use under national insurance in Japan before any other country in 2013^13,25^.

However, favorable results of additional intraoperative PDT using TPS can only be obtained if the cell population infiltrating the surrounding brain region can be efficiently targeted after resection of as much of the glioblastoma bulk as possible, because the intracerebral penetration depth of the irradiated laser light is limited to about 1 cm^26,27^. In other words, we believe that additional intraoperative PDT is indicated only for patients in whom sufficient surgical resection of the tumor can be achieved. Most glioblastomas originate in the cerebral hemispheres, mainly in the subcortical areas of these hemispheres, and the characteristic growth pattern of glioblastoma is to invade deep into the white matter. Hence, although there is the possibilities for adequate tumor removal in patients diagnosed at an early stage, once the tumor has invaded deep into the brain, it becomes difficult to remove more than 78%^5^ or more of the tumor to preserve brain function, and hence the chance to benefit from PDT, which we believe is the most effective treatment available also becomes low. Furthermore, about 8% of glioblastomas originate in the basal ganglia, such as the thalamus or in functional areas of the brain such as the brain stem, and there are cases in which surgery is limited to biopsy only for tissue diagnosis^28^. A recent summary of surgical procedures for glioblastoma in Japan showed that resection under craniotomy was performed in 78.7% to 79.3% of patients, whereas biopsy was performed in 14.3% to 15%^29^. According to the NCCN guidelines established in 2022^30^, for the patients with newly diagnosed glioblastoma that cannot be sufficiently resected, standard treatment is recommended only for patients younger than 70 years with a favorable performance status, and the physician's choice of the most favorable supportive care is recommended for patients who do not meet these criteria. A recent article reported the association between the extent of surgical resection and prognosis in 744 patients with *IDH* (isocitrate dehydrogenase) wild newly diagnosed glioblastoma showed that the prognosis of patients who underwent only biopsy was significantly less favorable than that of patients in whom substantial resection was performed, with a m-PFS of 5 months and a m-OS of 10 months^3^.

In this context, a group from Germany introduced the method of interstitial PDT (i-PDT), in which a fiber probe that can emit laser light is inserted into a tumor that is difficult to remove^31,32^. They used 5-aminolevulinic acid (5-ALA) as the PS, and performed PDT by puncturing the tumor with a laser irradiation fiber they had developed that can also be used for tissue biopsy^31^. Based on their technique, they reported the results of a clinical study of glioblastoma located in the unresectable regions^32,33^. The authors discussed that this i-PDT may solve one of the unmet medical needs in glioblastoma treatment, and we hence decided to investigate the possibilities of i-PDT using TPS.

In this article, we report the results of our histopathological analysis of the efficacy of i-PDT using TPS under diverse conditions in a mouse model of glioblastoma created by the subcutaneous implantation of C6 glioma cells, in which laser irradiation was performed by puncturing the tumor with a small-diameter plastic fiber probe that we developed. All methodologies, evaluations, and descriptions in this paper are based on the ARRIVE guidelines (http://arriveguidelines.org).

## Materials and methods

### Cell culture

The culture medium was prepared by adding 50 mL of heat-inactivated 10% fetal bovine serum (Gibco Thermo-Fisher Scientific, Waltham, MA, USA) and 50 U/mL penicillin–streptomycin solution (Sigma-Aldrich Co. LLC: St. Louis, MO, USA) to 500 mL of Dulbecco's Modified Eagle Medium (Gibco Thermo-Fisher Scientific). The C6 rat glioma cell line (Riken Cell Bank: Tsukuba, Ibaraki, Japan) was cultured in this culture medium in a 10-cm dish in a CO_2_ incubator (CO_2_ concentration 5%, 37 °C). The cells became confluent in about 3 days, and passages were repeated as necessary.

### Mouse allograft model

C6 glioma cells cultured in the above conditions were released and collected from the bottom of the dish by adding 1 mL of trypsin (Sigma-Aldrich Co. LLC: St. Louis, MO, USA), then centrifuged at 1000 rpm at 27 °C for 3 min. The supernatant was removed and the cells were diluted to 5 × 10^7^/mL with phosphate-buffered saline (PBS) to make the cell suspension for transplantation. The cell suspension (0.2 mL) was subcutaneously implanted into both lateral thighs of 6-weeks-old male thymus-deficient nude mice (BALB/c SLC-nu/nu, 20 g body weight, Japan SCL Inc., Hamamatsu, Shizuoka, Japan). The animals were reared normally for 2 weeks under specific-pathogen-free conditions and adjusted to have a tumor diameter of approximately 15 mm, and a body weight of about 27 g.

### Talaporfin sodium (TPS)

TPS (Laserphyrin®, Meiji Seika Pharma Co., Ltd., Chiyoda, Tokyo, Japan) is a PS with a molecular weight of 799.69. TPS is a hydrophilic compound that is synthesized by coupling chlorophyll and aspartic acid, and is considered a second-generation PS, with higher efficiency of reactive oxygen species (ROS) generation, and less side effects than with HpD which is considered a first-generation PS. TPS has an absorption band at about 664 nm, owing to its chlorin e6 moiety, which is a longer wavelength than that of HpD, and this increase the penetration depth of the irradiation light for its excition compared with HpD. TPS was approved for medical use in Japan, and has been used for the PDT treatment cancers such as early stage of lung cancer in 2003, malignant brain tumor in 2013 and recurrent esophageal cancer after radio-chemotherapy in 2014^13^.

### In vivo kinetics study of TPS

TPS diluted with physiological saline to various concentration (1 mg/mL, 5 mg/mL, 10 mg/mL, 15 mg/mL, 20 mg/mL, and 40 mg/mL) was administrated intraperitoneally to 6 groups of experimental model mice (5 mice per group), and then the accumulation kinetics of TPS in the subcutaneous tumors was analyzed over time. In vivo image analyzer system (IVIS), IVIS Lumina® (Caliper Life Sciences Hopkinton, MA, USA), was used to measure TPS. Near infrared extension 150 W tungsten EKE lamp was used as the excitation light and TPS accumulation was measured in the 605 to 630 nm wavelength range. The analysis software Living Image (Caliper Life Sciences) was used to set the ROI (region of interest) to the entire tumor, and average radiant efficiency (AREs: photons/sec/cm^2^/steradian [sr.]) were evaluated. Measurements were 15, 30, 45, 60, 90, 120, 150, 180, 210, 240, 300, 360 min after TPS administration. During the ARE measurement, the animals were anesthetized by isoflurane inhalation (Pfizer Inc., New York, NY, USA). The AREs obtained from the measurements were analyzed by one-way analysis of variance (ANOVA), and a *p*-value of less than 0.01was considered to indicate significant difference between groups.

### Treatment protocol for i-PDT using TPS

#### *Optical fiber for laser irradiation and jig for fiber fixation (*Fig. 1*)*

**Figure 1:**
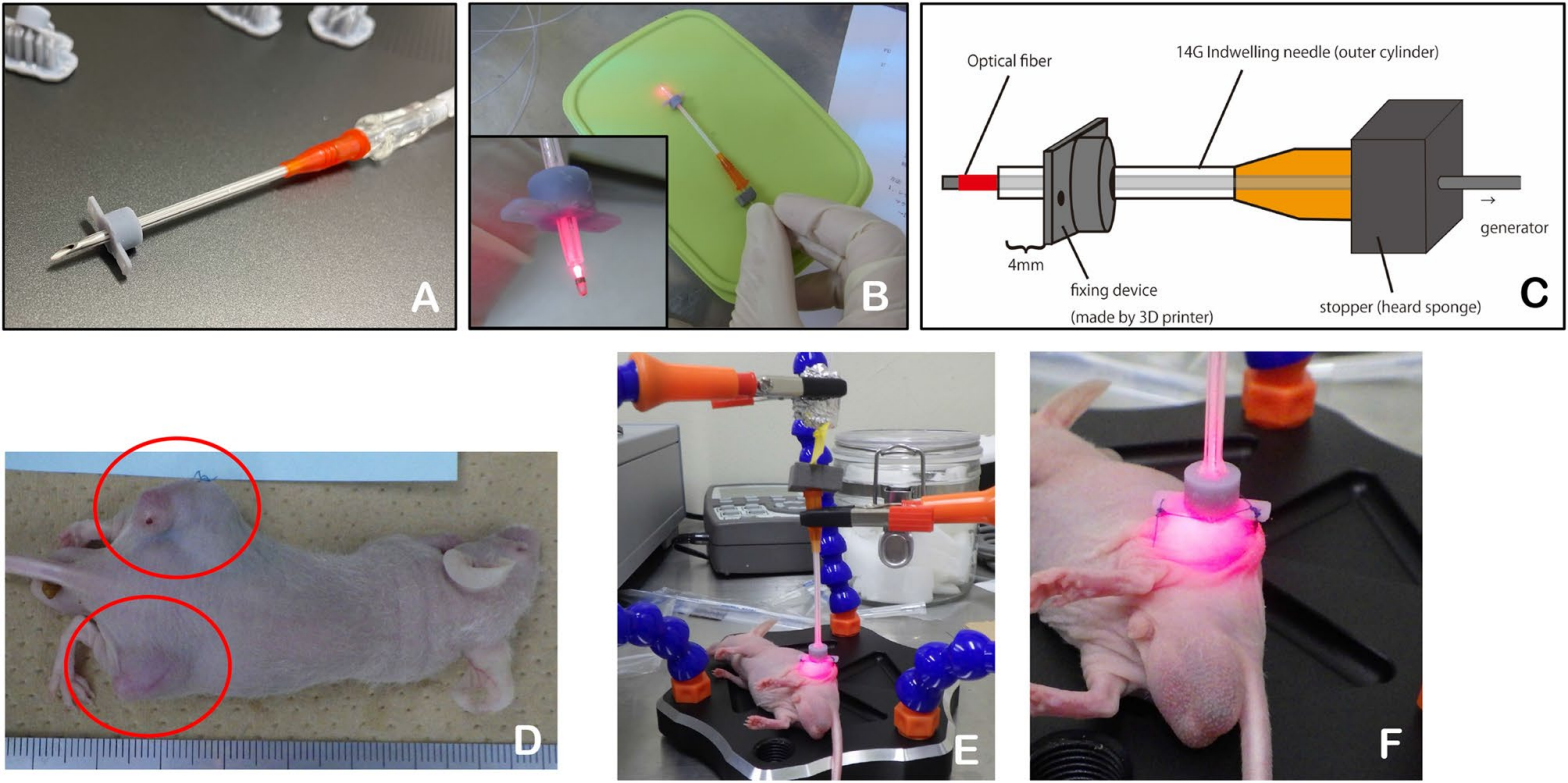
Devices used and method of i-PDT. (**A**–**C**) The i-PDT unit developed by the authors. (**D**) C6 glioma cells were subcutaneously implanted into the bilateral thighs of mice (circled areas). (**E**,**F**) A mouse undergoing i-PDT for a subcutaneous tumor. A jig to precisely guide the light irradiation with small-diameter optical fiber to the center of the tumor and to stabilize the fiber position. The optical fiber was fixed precisely to the center of the subcutaneous tumor and irradiated 664 nm laser light.

The authors developed an original small-diameter optical fiber for irradiation to perform i-PDT. Specifically, it is an optical fiber made of polymethyl methacrylate (diameter 0.8 mm: emission length 2.0 mm: emission area 0.0553 cm^2^, transmittance 65%: Nissei Electric Co., Machida, Tokyo, Japan). The light source was a Rouge-LD (664 nm, 500mW, class 4 continuous wave laser diode: Cyber Laser Inc., Minato, Tokyo, Japan). In addition, the authors developed an original jig to precisely guide the light irradiation with a small-diameter optical fiber to the center of the tumor and to stabilize the fiber position. Specifically, the jig has a fixation wing created using a 3D printer for suture fixation to the skin of a mouse thigh which can fit inside the outer casing of a 14-gauge vein needle. The optical fiber itself is equipped with a stopper to ensure that the luminescent surface is exposed at a precise distance from the outer cylinder of the indwelling needle.

#### Irradiation method

The irradiation targets were the subcutaneous tumors int the left and right thigh of the transplanted mice with a diameter of approximately 15 mm. Experimental model mice were administered 10 mg/kg of TPS intraperitoneally, and 90 min later, a puncture needle with the aforementioned 14-gauge outer tube was sharply inserted into the center of the tumor, after which the inner tube needle was removed and the outer tube was used as an insertion guide for the optical fiber. The optical fiber was fixed precisely so that it was buried in the midline of the tumor with a length of up to 4 mm from the tip of the fiber, and the actual emission was cylindrical 2 mm long emission of 664 nm laser light (Fig. 1).

#### Irradiation conditions

To analyze the difference in the effect of i-PDT depending on the conditions under which PDT is performed, the following 6 groups of irradiation conditions were used, with 5 mice in each group. Group A and B were the low power density and long time irradiation groups: Group A (150 mW/cm^2^, 50 J/cm^2^, 512 s) and Group B (150 mW/cm^2^, 100 J/cm^2^, 1025 s). Group C and D were the high power density and short time irradiation groups: Group C (758.65 mW/cm^2^, 50 J/cm^2^, 100 s) and Group D (768.65 mW/cm^2^, 100 J/cm^2^, 200 s). Group E (3852 mW/cm^2^, 500 J/cm^2^, 200 s) was ultrahigh power density and shorter time irradiation. The control, Group F was high power and shorter time irradiation (768.65 mW/cm^2^, 100 J/cm^2^, 200 s) without the administration of TPS and Group G was no irradiation with only the administration of TPS (Table 1).

**Table 1:** Conditions for performing i-PDT in each group.

	Energy density to tumor (J/cm^2^)	Total energy density (J/cm^2^)	Power density (mW/cm^2^)	Input power (mW)	Time (s)	Injection of TPS
A	50	76.95	150	8.3	513	+
B	100	153.75	150	8.3	1025	+
C	50	77.05	385.23	21.3	200	+
D	100	153.73	768.65	42.5	200	+
E	500	772.45	3852.27	213	200	+
F	100	153.73	768.65	42.5	200	−
G	0	0	0	0	0	+

Group A through E were groups that underwent PDT under various conditions. The control, Group F was the group that underwent laser irradiation without the administration of TPS, and Group G was no irradiation with only the administration of TPS.

*TPS* talaporfin sodium.

### Post irradiation specimen preparation

At 1 and 3 h after PDT, experimental mice were sacrificed by cervical dislocation under anesthesia with intraperitoneal Somnopentyl® (Kyoritsu Seiyaku Co., Chiyoda, Tokyo, Japan) administration, and subcutaneous tumors were removed and fixed by immersion in 10% neutral formalin solution with the outer tube of the intravenous needle attached. After fixation, the tumor tissue was split in the midline so that the irradiated surface could be seen, paraffin-embedded, and then 6-μm thin sections of were prepared using microtome.

### Histopathological examination

#### Light microscopic examination

Tissue sections were subjected to hematoxylin eosin (HE) staining for morphological analysis of the tissue. For the evaluation of i-PDT-induced tissue changes, in addition to the morphological changes in the tumor tissue, the area of tissue changes that may be a result of i-PDT was measured using NDP.view2 software (Hamamatsu Photonics K.K., Hamamatsu, Shizuoka, Japan). For evaluation of the tumor-killing effects of i-PDT, the TUNEL method (in situ apoptosis detection kit MK500, Takara Bio, Japan) was used in which the label was a rabbit-derived anti-fluorescein isothiocyanate (FITC) horseradish peroxidase (HRP) conjugate and 10 × diluted terminal nucleotidyl transferase, and the chromogenic substrate was a 50 × diluted 3,3'-diaminobenzidine (DAB) solution. DAB staining was performed for nuclear staining. The effect of i-PDT on tumor blood vessels was analyzed by phosphotungstic acid hematoxylin (PTAH) staining for the presence of intravascular thrombi and vascular endothelial injury was analyzed by immunohistochemical staining with anti-CD31 antibody (Dianova, Biozol Co., Hamburg, Germany).

#### Electron microscopic analysis

The excised tissues were quickly fixed with a 4% paraformaldehyde/2.5% glutaraldehyde mixture (pH 7.2 in 10 mM PBS) followed by post-fixation with 2% osmium tetroxide solution for 50 min. Next, dehydration was performed with 30%, 50%, 80%, and 100% ethanol for 10 min each. Then tissues were incubated in n-butyl glycidyl ether (QY-1, EM Japan Co. Ltd. Bunkyo, Tokyo, Japan)-anhydrous ethanol (1:1 ratio) for 15 min, then in 100% QY-1 for 15 min twice, and then treated with epoxy resin solution (Quetol-812, EM Japan Co. Ltd. Bunkyo, Tokyo, Japan): QY1 (1:3, 1:1, 3:1 ration for 15 min each) for epoxy resin replacement. The specimens were treated with 100% epoxy resin solution for 12 h and then at 60 °C for 3 days, then sliced into ultra-thin section using an ultramicrotome, double-stained with uranium acetate + lead nitrate staining solution, and observed using a transmission electron microscope (JEM-1400 Flash, JEOL Ltd., Akishima, Tokyo, Japan).

### Statistical analysis

All data were analyzed using Microsoft Excel for Microsoft 365 MSO 64bit version 2202 (Microsoft Corporation, Redmond, WA, USA) and presented as the mean ± standard deviation. Analysis of the tumor accumulation of TPS by IVIS, the area of the tumor-killing effect, and the residual tumor area after PDT at various energy densities were compared between the groups to determine significant differences. One-way analysis of ANOVA was used for statistical analyzes, and a *p*-value of less than 0.01 was considered to indicate a significance difference between groups.

### Ethics

This study was approved by the Ethics Committee for Animal Experiments of Tokyo Medical University (project approval no.: R3-0096). In addition, all methods were performed in accordance with the relevant guidelines and regulations.

## Results

### In vivo kinetics of TPS

**Figure 2:**
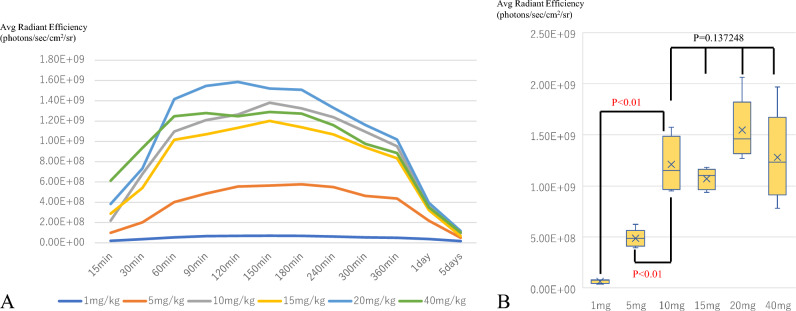
In vivo kinetics of TPS. (**A**) Degree of tumor uptake over time for each administration dose of TPS. (**B**) Tumor fluorescence at 90 min after the administration of each dose of TPS. The average radiant efficiency of TPS from subcutaneous tumors identified by the IVIS system peaked at 90 min after administration, regardless of the concentration of TPS administered. The average radiant efficiency showed a significant difference after an intraperitoneal dose of 10 mg/kg.

Regardless of TPS dose, all groups showed a temporal increase in fluorescence intensity after the intraperitoneal administration of TPS, which peaked at 90 min (Fig. 2A). Thereafter, high fluorescence values were maintained until 120 min, then gradually decreased to 29% of the peak level at 24 h and 7% at 5 days. Peak values at 90 min after TPS administration were significantly higher in the 10 mg/kg than in the 1 mg/kg and 5 mg/kg groups (one-way ANOVA, *p* < 0.01), but there was no significant difference between the 10 mg/kg, 15 mg/kg, 20 mg/kg, and 40 mg/kg groups (Fig. 2B). Based on these results, we concluded that the optimal intraperitoneal dose of TPS in this experiment of i-PDT was 10 mg/kg and the optimal timing of irradiation was 90 min after TPS administration.

### Efficacy of i-PDT using TPS

#### Comparative analysis of tissue injury levels

**Figure 3:**
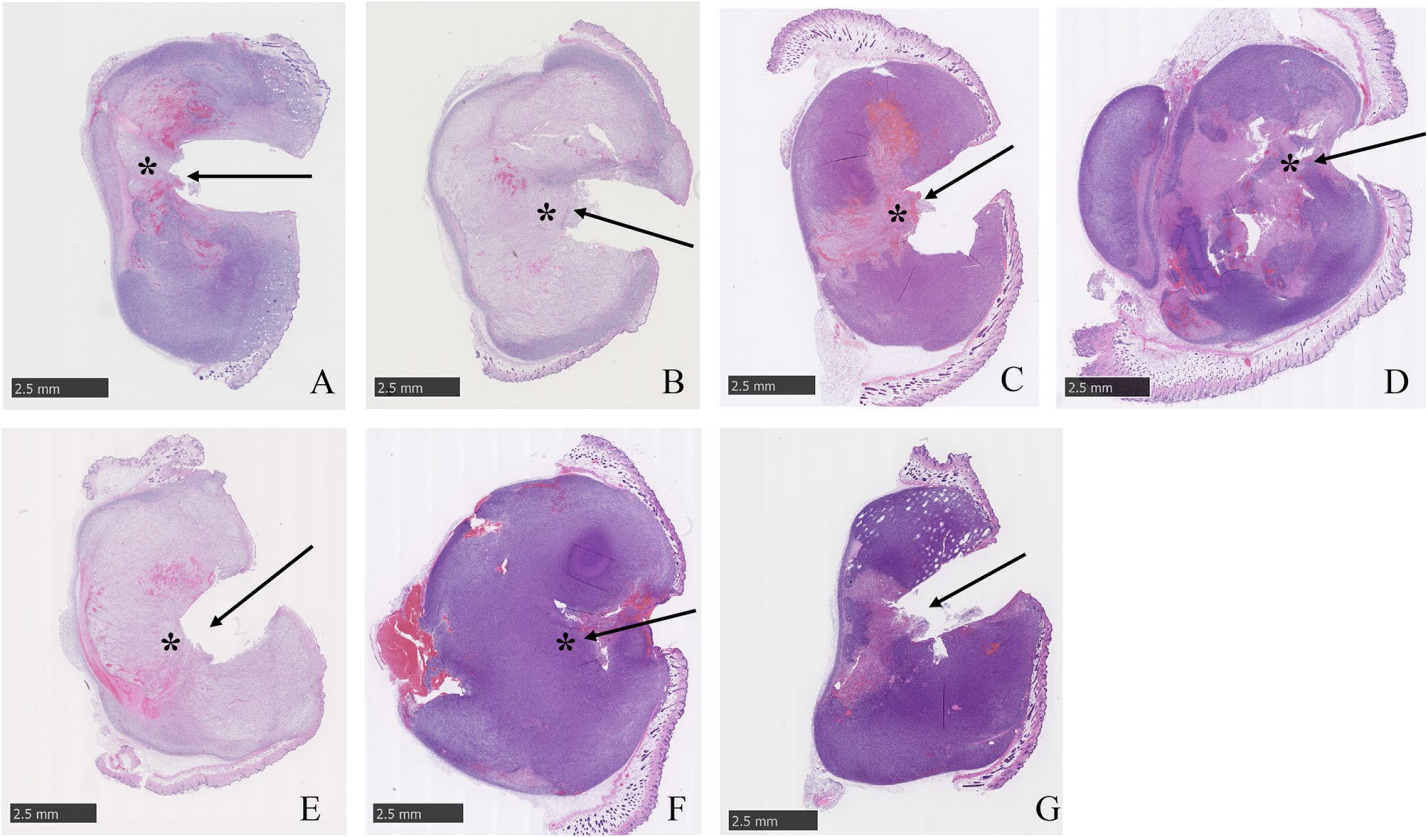
Tumor histology after i-PDT for various irradiation conditions. (**A**–**E**) Various irradiation conditions, and Controls (**F**,**G**). (Table 1). Asterisk: location of light source, Arrow: direction of fiber insertion. The area of tumor killing effect by i-PDT was significantly larger at 100 J/cm^2^ (**B**,**D**) than 50 J/cm^2^ (**A**,**C**). At the same energy density of 100 J/cm^2^, the i-PDT effect was larger, (**B**) when the power density was lower (150 mW/cm^2^) and the irradiation time was longer (1025 s) than (**D**), when power density was 768.65 mW/cm^2^ and the irradiation time was 200 s.

**Figure 4:**
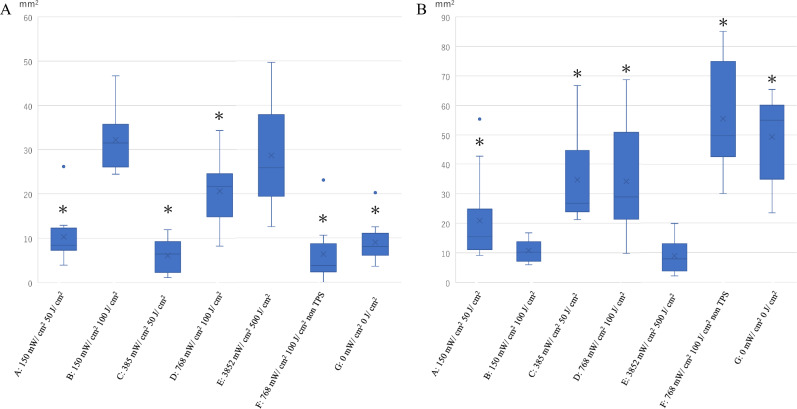
Tumor tissue injury area (**A**) and residual area (**B**) for each irradiation condition. The group with asterisk shows a significant difference (p < 0.01) in necrotic area and morphologically viable area compared to group B, which showed the best PDT effect. Of particular note is that there was no significant difference between Group E, which were irradiated at very high power and energy densities.

We were able to perform the i-PDT technique stably in all groups. Average values of the PDT effects and the area of the residual tumor were measured in 10 sections from each group. In the case of laser irradiation at a power density of 150 mW/cm^2^, when the energy densities were 50 J/cm^2^ and 100 J/cm^2^, the area of the tumor killing effect by i-PDT was significantly larger at 100 J/cm^2^, and the residual tumor area was significantly smaller for the 100 J/cm^2^ group (*p* < 0.01). At the same energy density of 100 J/cm^2^, the i-PDT effect area was larger and the residual tumor area was smaller when the power density was lower and the irradiation time was longer (*p* < 0.01). Similar results were observed at an energy density of 50 J/cm^2^, but no significant difference was observed. In addition, a comparison of the ultrahigh power density (3852 mW/cm^2^, 500 J/cm^2^) group and the power density 150 mW/cm^2^ and energy density 100 J/cm^2^ group showed no significant difference in i-PDT effect area and residual tumor area (Figs. 3 and 4).

#### Changes in i-PDT effects over time

**Figure 5:**
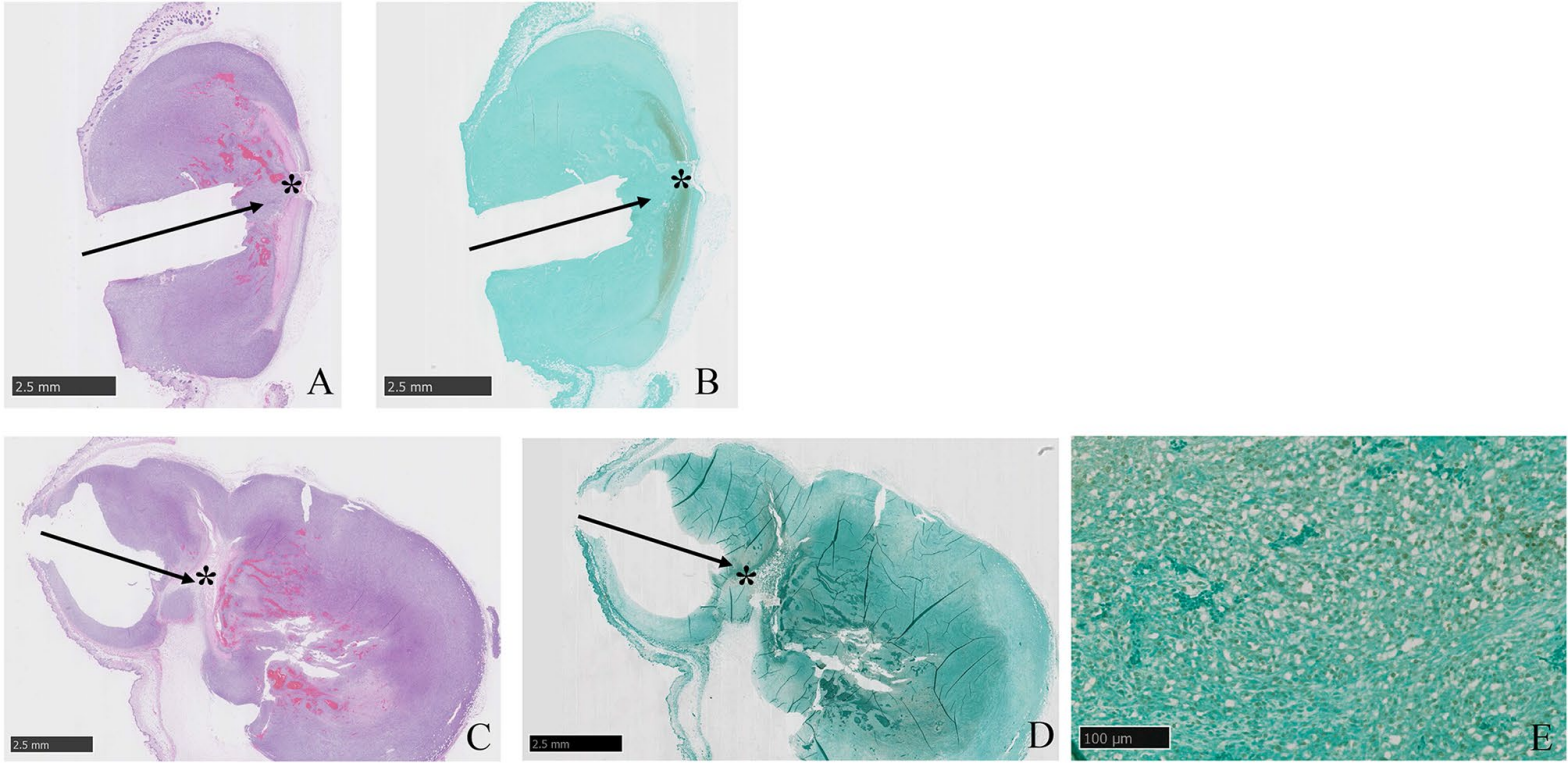
Histological findings at 1 h (**A**,**B**) and 3 h (**C**–**E**) after i-PDT. (**A**,**C**) HE staining, (**B**,**D**,**E**) TUNEL method. Asterisk: location of the light source, Arrow: direction of fiber insertion. The degree of tissue damage caused by i-PDT did not differ at 1 and 3 h after i-PDT. In both timings, tissue necrosis was observed in the area close to the light source, and TUNEL-positive apoptosis was seen in the area a little further away from the light source.

In the 1-h post i-PDT group, the PDT effect was limited to the area around the light source, and the intracellular changes were limited to nuclear aggregation. In the 3-h post i-PDT group, the extent of tissue damage was similar, but progression of tumor cell damage, including the appearance of ghost cells and TUNEL-positive cells, was observed (Fig. 5).

#### Histopathological changes by i-PDT

**Figure 6:**
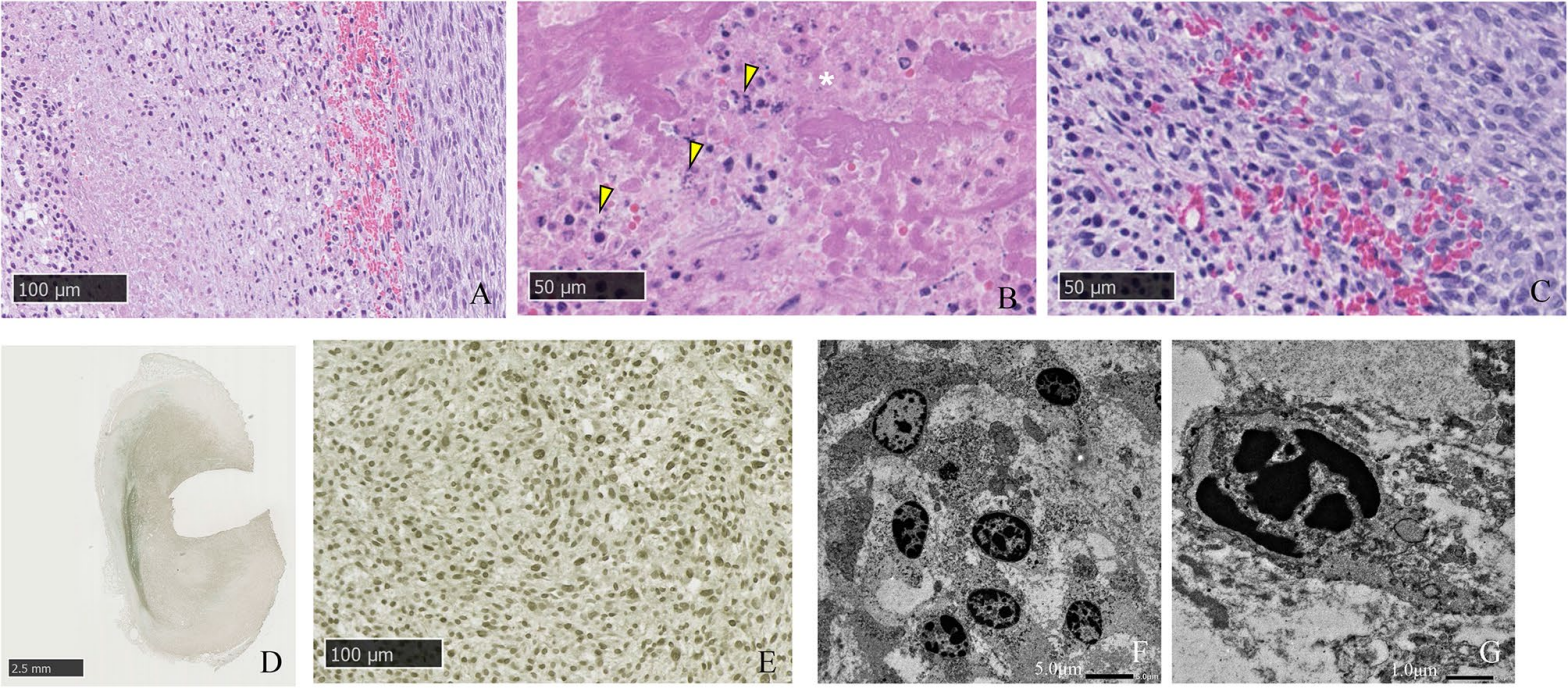
Pathological findings of tumor tissues in the 150 mW/cm^2^ and 100 J/cm^2^ irradiation groups. (**A**–**C**) HE staining, (**D**,**E**) TUNEL method, (**F**,**G)** electron microscopic findings. Asterisk: ghost cells, Yellow arrow head: apoptotic tumor cells. The area near the light source was found to be a layer of ghost cells, and a layer of tumor cells with condensed nuclei and cytoplasm could be seen on the outside of the layer. The nuclei of the cells in this area were TUNEL-positive, and showed chromatin aggregation and fragmentation.

HE staining showed a band of denucleated ghost cells in the tumor tissue around the irradiation fiber, which was the light source, and many apoptotic bodies with disrupted nuclei and a rounded and distended cytoplasm were observed. In tissues slightly distant from the light source, tumor cells showed only cytoplasmic swelling and nuclear aggregation, and further away from the light source, tumor cell morphology tended to be unaffected. The TUNEL method showed a strong nuclear staining in the same region where the HE staining showed tumor-killing effect, suggesting that apoptosis had occurred. Electron microscopic observation of the area where the cell-killing effect was observed small circular nuclei, aggregation of intranuclear chromatin, and fractionation of the nuclei (Fig. 6).

**Figure 7:**
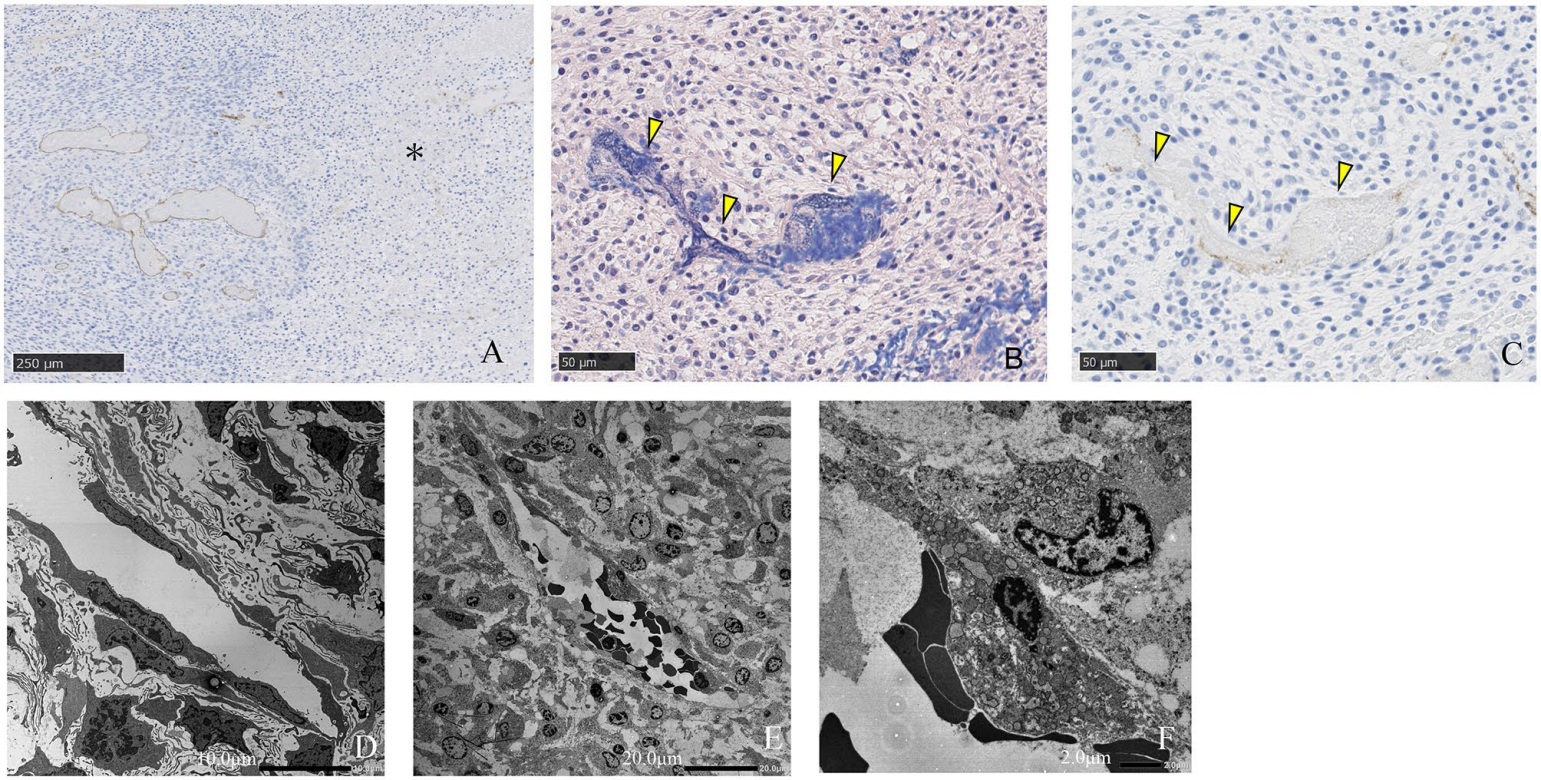
Pathological findings of intra tumoral micro-vessels in the 150 mW/cm^2^ and 100 J/cm^2^ irradiation groups. (**A**,**C**) Immunohistochemical staining using an anti-CD31 antibody, (**B**) PTAH staining, (**D**–**F**) electron microscopic findings. Asterisk: the area of ghost cells, Yellow arrow head: micro-vessels in the layer of apoptotic tumor cells. Tumor micro-vessels in the layer of apoptotic tumor cells was filled with PTAH-positive fibrin thrombi, and had also lost their CD-31 expression. (**A**–**C**) Endothelial cells of the micro-vessels in this layer (**E**,**F**) demonstrated degeneration compared with the viable tumor cell layer (**D**).

Immunostaining with an anti-CD31 antibody showed that staining of vascular endothelial cells in intra tumoral micro-vessels was maintained in the residual tumor area, but in areas where the cell-killing effect of i-PDT was observed, staining of vascular endothelial cells with this anti-CD31 antibody was reduced. PTAH staining of the same area showed numerous fibrin-based thrombus formations in the vessels, with reduced anti-CD31 antibody staining. Electron microscopic images of tissue subjected to i-PDT showed findings suggestive of vascular endothelial damage, including the aggregation of nuclear chromatin, vacuole formation in the swollen cytoplasm, and shortened projections in the vascular endothelial cells compared with tissue not subjected to i-PDT (Fig. 7).

## Discussion

The tumor-killing effect of PDT on cancer cells is exerted through a complex mechanism of direct cellular damage caused by singlet oxygen generated by the excitation of a PS accumulated within tumor cells, as well as indirect effects such as intimal damage in intra-tumoral micro-vessels, embolic mechanisms caused by blood stagnation owing to the spasm of arterioles, and inflammatory responses by immune cells^14,34,35^. In this study, i-PDT-induced cell damage such as the formation of ghost cells, cytoplasmic swelling, and nuclear fragmentation were observed around the laser irradiation source, indicating apoptosis. On the other hand, from the area about 2 mm away from the irradiation source, the changes were limited to cytoplasmic shrinkage and aggregation of nuclear chromatin. Immunostaining of the site with anti-CD31 antibodies to detect endothelial cells in the intratumor vessels was reduced, and electron microscopy images showed evidence of endothelial cell degeneration. Furthermore, PTAH staining confirmed the formation of fibrin thrombi in the vascular lumen. From these results, it was suggested that tumor tissue damage by i-PDT is mainly caused by singlet oxygen-induced apoptosis of tumor cells in the proximal region of the irradiation source, and that ischemic cell damage and vascular shutdown effects owing to embolic mechanisms of intra-tumor vessels occur in addition to apoptosis in the slightly distant regions.

The direct tumor-killing effect of PDT is thought to occur when TPS is selectively taken up by the lysosomes of tumor cells, and the energy generated when the accumulated TPS is excited by laser energy and returns to a steady state converts dissolved oxygen in the tissue into highly toxic singlet oxygen, resulting in activation of the programmed cell death (PCD) pathway in tumor cells, i.e., PCD types 1 and 2 apoptosis and autophagy-like tumor-killing effects^14,34,36^. Miki. et al.^23^ demonstrated that PDT using TPS to T98G glioblastoma cells induced a concentration-dependent increase in caspase 3 activity, the main pathway of apoptosis, and flow cytometry using annexin V-FITC confirmed the induction of apoptosis^23,36^. On the other hand, Kushibiki. et al.^37^ reported that PDT activates lysosomal hydrolytic enzymes, which induces mitochondria-mediated apoptosis by transforming Bid, a Bcl-2 family protein, into active t-Bid. This activation of the apoptotic pathway by crosstalk of lysosomes and mitochondria is thought to be the main mechanism of the cytotoxic effects of PDT on cancer^36^.

The present study noted tissue changes at 1 and 3 h after PDT, showing that the extent of the cell-killing effect remains the same, but changes at the cellular level progress with time. Using an intracerebral implanted model of the C6 glioma cell line, Namatame. et al^22^. showed that M30 cyto-DEATH, an apoptosis-associated protein, was increased time dependent in the tumor tissue at 1, 3, and 6 h after PDT from the brain surface using TPS^22^. However, as in the present study, no increase in the extent of tissue injury was observed. Research should be conducted in the future to determine the limit of single irradiation by observing tissue changes over a long period of time, as well as simulation studies of light delivery to determine the effective range of i-PDT.

On the other hand, previous studies have reported the vascular shutdown effect of PDT using TPS. Moy. et al.^38^ stated that in an experiment in which PDT was performed on normal vessels in the skin of mice treated with TPS, permanent blood flow interruption occurred at energy densities of 85 J/cm^2^ or higher^38^. They noted that no change in the appearance of the vessels was observed, and only blood flow was interrupted. McMahoon. et al.^39^ investigated the effects of PDT with TPS on micro-vessels of normal cremaster muscle in rats and found that laser irradiation at 75 mW/cm^2^ and 135 J/cm^2^ caused the stagnation of blood flow owing to platelet thrombus formation^39^. They also found that the degree of blood flow impairment caused by PDT depends on the time lapse between TPS administration and PDT and the dose of TPS administered, as well as the effect of PDT on cancer^39^. In the experimental system of our present study, the TPS dose and the timing of i-PDT application were determined from the results of a kinetic study that analyzed the state of TPS accumulation in tumor tissue. As a result, we fixed the intraperitoneal dose of TPS in mice at 10 mg/kg and the optimal timing between TPS administration and i-PDT was set at 90 min. Therefore, the cytotoxic effect and vascular shutdown effect of i-PDT depended exactly on the conditions of the laser irradiation. As shown in Figs. 3 and 4, the same irradiation power density of 150 mW/cm^2^ resulted in more extensive tumor tissue damage with an irradiation energy density of 100 J/cm^2^ than with that of 50 J/cm^2^ (Figs. 3A,B and 4A,B). According to Moy's theory mentioned above^38^, the vascular shut down effect does not occur at 50 J/cm^2^ of irradiation, and therefore, it can be inferred that the cytotoxic effect of 100 J/cm^2^ irradiation on tumor cells and ischemic tissue damage owing to vascular shutdown are additive outcomes.

The most interesting findings in this study were that for the same irradiation energy of 100 J/cm^2^, an irradiation power density of 150 mW/cm^2^ showed a stronger i-PDT effect than a power density of 768 mW/cm^2^ (Fig. 4A,B). In other words, irradiation for a long time at a low power density induces more extensive tumor tissue damage and a significantly smaller residual tumor area than irradiation for a short time at a high power density. In an experiment of PDT by surface irradiation using a subcutaneous transplantation model of lung cancer tumor cells, Kawakubo et al.^40^ reported that in the high irradiation power group, there was a layer in the subcutaneous tumor surface area where no PDT effect was observed^40^. They also evaluated epidermal blood flow and found that immediately after the high-power density irradiation group, blood flow in the tumor nutrient vessels of the epidermis was reduced by half owing to vascular shutdown, resulting in a reduced oxygen supply to the tumor^40^. Tetard et al.^41^ who performed 5-ALA PDT in a rat brain tumor transplant model also reported that low-power density irradiation produced a higher PDT effect than the high-power density irradiation. They also stated that the reason is the reduced oxygen supply to the tumor tissue owing to vascular shutdown caused by high-power density irradiation, and that it is more favorable to perform PDT intermittently (metronomic PDT) and wait for oxygen to be resupplied during the pause in PDT than to perform continuous PDT^41^.

In our present i-PDT experiments, a laser light source was inserted into the center of the tumor. The center of the tumor is a site where nutritional supply is not adequate for the growth of the tumor and hence central necrosis is likely to occur. Therefore, it is presumed that inducing further oxygen deprivation by administering PDT would further diminish its effectiveness. Therefore, in i-PDT, which is performed by puncturing the laser source into the midline of the tumor, it is essential to perform the procedure at a low energy density and over a longer period of time, and at an irradiation energy density of 100 J/cm^2^ or higher. In conclusion, we speculate that the key to successful i-PDT lies in striking a balance between the cytotoxic effect and the vascular shutdown effect, and in setting the probe position considering effective light delivery^42^.

Limitations of this research include: the use of thymus-deficient immunodeficient mice did not allow us to examine the third cell-killing mechanism of PDT, the analysis of the results was limited to pathomorphological examination and the analysis time is short, up to 3 h after i-PDT, the study was small (5 mice per group), and the experimental methods were conducted solely by our group and not verified by others.

In the future, to confirm the long-term effects of PDT, we are planning to develop the experimental methods that takes into account the immune response^35^. In addition, to clarify the effects of PDT on brain tumors, it is essential to perform analyses in a brain transplant model. Such an experimental system is expected to be more difficult to control the conditions for i-PDT by drug delivery than the subcutaneous transplantation model, owing to the involvement of the blood–brain barrier, the distribution of oxygen in the tumor tissue, and the specific characteristics of light delivery. However, we plan to carefully design our future experimental systems, to obtain clear i-PDT effects similar to the present study.

## Conclusion

After intraperitoneal administration of the second-generation photosensitizer, TPS, to a glioblastoma mouse model with subcutaneously implanted C6 glioma cells, i-PDT experiments were performed using a 664 nm laser beam that can excite TPS via a small-diameter plastic fiber probe punctured into the tumor center. As a result, an irradiation energy density of 100 J/cm^2^ or higher was required to obtain a sufficient therapeutic effect on tumor tissue with a 15-mm diameter. In the area close to the light source, the cell damage was mainly apoptotic cell death, which is considered to be a direct effect of PDT. However, in the area slightly distant from the light source, in addition to apoptotic cell damage, a vascular shunt down effect mainly caused by fibrin thrombi was observed. In addition, when performing i-PDT at the same irradiation energy density, it appeared useful to perform irradiation at a low power density for a long period of time, to suppress the vascular shutdown effect and maintain the oxygen supply to the tumor.

## Data Availability

The datasets used and/or analyzed during the current study available from the corresponding author on reasonable request.
